# Modelling 3D Tumour Microenvironment In Vivo: A Tool to Predict Cancer Fate

**DOI:** 10.3390/cimb45110569

**Published:** 2023-11-14

**Authors:** Johanna Marines, Francesca Lorenzini, Karima Kissa, Laura Fontenille

**Affiliations:** 1 AZELEAD, 377 rue du Pr Blayac, 34080 Montpellier, France; jmarines@laposte.net (J.M.); francesca.lorenzini@unitn.it (F.L.); 2 Molecular Basis of Inflammation Laboratory, Institut de Génétique Humaine, CNRS, Université de Montpellier, 34090 Montpellier, France; 3 VBIC, INSERM U1047, Université de Montpellier, 34090 Montpellier, France

**Keywords:** cancer, microenvironment, live imaging, PDX, zebrafish

## Abstract

Recently, many studies demonstrated the fundamental role of the tumour microenvironment (TME) in cancer progression. Here, we describe a method to visualize in 3D the behaviour of tumours in zebrafish embryos. We highlight two major actors of the TME, macrophages and vessels. This valuable tool is transposable to Patient-Derived Xenograft imaging in order to predict the fate of malignant tumours according to the dynamics of their TME.

## 1. Introduction

TME characterisation has become over the years a major topic in the understanding of tissue tumorigenesis and the development of treatments. The tumour niche is a very dynamic region where different cell types coexist and interact with cancer cells and condition their fate [1]. Among them, vessels play an important role as a pathway for cancer dissemination [2] and, at the same time, for oxygen and nutrient supply [3]. Similarly, the recruitment and infiltration of several types of immune cells in the tumour such as macrophages, neutrophils and lymphocytes have been described as having a direct impact on cancer progression [4]. Finally, the composition of the tumour niche is nowadays used as a means of diagnosis and an indicator of good or bad prognosis [5,6]. Therefore, the 3D dynamic visualization of the tumour microenvironment appears as a predictive tool of cancer cell behaviour in terms of intravasation, invasiveness and metastasis. To this end, extensive efforts are currently being made to explore 3D reconstruction of human cancer biopsies [7,8]. However, to study in detail the molecular and cellular mechanisms of cancer progression, innovative methods based on in vivo models are essentials.

The use of in vitro immortalized cell lines has been the gold standard for many years. However, 2D and 3D culture models remain limited by the number of co-cultured cell types which do not sufficiently account for the complexity of the TME [9]. Thus, in vivo models are crucial to reliably reproduce the interactions between different cell types. In recent years, zebrafish embryos have emerged as a relevant tool in oncology. The simple manipulation, the low cost of rearing and the wide range of genetic manipulations make them one of the most pertinent tools for cancer in vivo studies. In addition, their transparency allows for imaging and visualization in real time of the conserved cellular processes involved in tumour dissemination, as well as the interactions between cancer cells and their microenvironment [10]. Xenografts of human cancer cells in zebrafish embryos have been widely used to model different types of cancer [11]. Indeed, due to the immaturity of their adaptive immune system at the embryonic stage, rejection after transplantation of human cancer cells is prevented [12]. Over the last decades, several teams have characterized the types of interactions between xenografted human cancer cells and macrophages in the zebrafish embryo [13,14]. Furthermore, the interaction between cancer cells and vessels has been intensively studied. Processes such as angiogenesis and vessel co-option reported in patients are conserved after xenografting glioblastoma and melanoma into the zebrafish embryo [15,16]. The feasibility of simultaneous imaging of three different populations in the yolk sac using two zebrafish reporter lines has been shown [17]. However, 3D and 4D visualization of macrophages and surrounding vessels in the TME has never been modelled.

## 2. Materials and Methods

### 2.1. Animal Rearing

Adult zebrafish (*Danio rerio*) were maintained on a 12/12 h light/dark cycle in a partially recirculating system and fed 3 times/day with fresh Artemia salina and dry food. Outcross of homozygous transgenic zebrafish lines *tg (KDR:eGFP)* and *tg (mfap:RFP)* were used to perform cell transplantations. All experimental procedures on zebrafish were performed in accordance with the European guidelines and regulations on Animal Protection from the French Ministry of Health (F341725).

### 2.2. Cell Culture Maintenance

Human glioblastoma cancer cell line, U87 WT, was purchased from ATCC (U-87 MG ATCC^®^ HTB-14™). Human melanoma cancer cell line, A375 WT, was kindly provided by A. Pelegrin from IRCM—Montpellier. U87 and A375 cells were cultured in DMEM (Eurobio scientific, Les Ulis, France) supplemented with 1% L-glutamine 200 mm (Eurobio scientific, Les Ulis, France), 1% Penicillin/Streptomycin (Eurobio scientific #CABPES01-0U, Les Ulis, France) and 10% foetal bovine serum (Eurobio scientific, Les Ulis, France) at standard conditions of 5% CO_2_, at 37 °C. Cells were cultured in 100 mm Petri dish (Corning, Corning, NY, USA) and split twice a week when they reached 80–90% confluency.

### 2.3. Generation of Stable Fluorescent Human Cancer Cell Lines

To obtain U87-eBFP+ (blue fluorescent protein) and A375-eBFP+ cells, U87 WT and A375 cells were stably transfected with EBFP2-N1 plasmid using Phosphate calcium. EBFP2-N1 was a gift from Michael Davidson (Addgene plasmid #54595). One day before transfection, cells were plated in 6 well plates. EBFP2-N1 purified plasmid (4 µg) was transfected using 2.5 M CaCl_2_ reagent. At 24 h after transfection, complete DMEM medium was replaced. eBFP+ cells were selected using Geneticin (800–1000 µg/mL) diluted in DMEM media during 4–6 weeks before starting in vivo experiments.

### 2.4. Embryo and Cell Preparation Prior to Xenotransplantation

Embryos were initially maintained at 28 °C at a maximum density of 50 embryos per Petri dish in fish water supplemented with 0.0002% methylene blue (Sigma, Burlington, MA, USA) as an antifungal agent. After 24 h, embryos were placed in fish water containing 200 µM Phenylthiourea (PTU) to prevent embryo pigmentation and allow fluorescent imaging acquisition.

Glioblastoma cells (U87-eBFP+) and melanoma cells (A375-eBFP+) were harvested from a 100 mm Petri dish on the day of xenograft transplantation. Cells were washed with phosphate-buffered saline (PBS) and detached using Trypsine-Versene EDTA (Eurobio scientific) at 37 °C for 5 min. Trypsine-versene was inactivated by complete DMEM media, and cells were pelleted (1000 rpm–5 min) then washed with PBS. Finally, cells were resuspended in 50 µL PBS to ensure highly concentrated cell preparation.

### 2.5. Xenotransplantation of Human Cancer Cell Lines

Borosilicate glass capillaries (O.D.: 1 mm, I.D.: 0.75 mm, Sutter Instrument, Novato, CA, USA) were pulled using glass micropipette puller (Sutter Instrument, Novato, CA, USA). Capillaries were filled with 10 µL of cells suspension. Injection was performed under a stereomicroscope (Leica M80 Stereo zoom microscope) using a micromanipulator (Narishige, London, UK) and oil manual microinjector (Cell Tram Vario, Eppendorf, Hamburg, Germany). Before xenografting, embryos were anesthetized in 0.16 mg/mL PBS/Tricaine (MS-222) then tidily aligned laterally for melanoma or dorsally for glioblastoma transplantation. Embryos were maintained in position during injection using forceps. Melanoma cells were transplanted in the swim bladder, while glioblastoma cells were transplanted in the midbrain region. Between 50 and 100 cells were transplanted per embryo. Transplanted embryos were maintained at 33 °C in fish water containing PTU.

### 2.6. The 3D and 4D Live-Imaging Acquisition

A fluorescence stereomicroscope (AXIO Zoom.V16—Zeiss, Oberkochen, Germany) equipped with eBFP filter was used for phenotypic selection of xenotransplanted embryos. Embryos with fluorescent tumour mass in the swim bladder or in the brain were selected for image/time lapse acquisition. 4 or 5 dpf (1- or 2-days post transplantation) anaesthetized embryos were mounted laterally or dorsally in 0.7% low melting point agarose (Life Technologies, Carlsbad, CA, USA) in a 35 mm fluorodish covered with fish water containing Tricaine. Images and time lapse were acquired at 33 °C using a Confocal spinning disk ANDOR coupled to a Nikon Ti Eclipse microscope (Objectives: 20×/0.75). The 405/488/561 lasers were used with appropriate filters. For time-lapse, images were taken each 7 min with 1 µm z-step interval.

### 2.7. Image Analysis

Images and time lapse were processed using Fiji (version 2.9.0/1.53t) and Imaris software (version 9.5). Images were smoothened and brightness/contrast was adjusted using Fiji software over all Z-stacks. Maximum projections were generated using Fiji software. Segmentation and 3D reconstructions of macrophages, vessels and cancer cells were performed using Imaris Software. Supplementary videos were recorded using the same software.

## 3. Results

### 3.1. Cell Lines Engineering, Transplanted into Zebrafish Embryos and Live Imaging

In order to follow and predict the tumour’s fate, fluorescent human glioblastoma (U87) and human melanoma (A375) cell lines were engineered and transplanted into the midbrain and swim bladder of early-stage zebrafish embryos. Using two zebrafish transgenic reporter lines, *Tg (kdrl:gfp)* and *Tg (mfap:RFP)*, we were able to simultaneously study the behaviour of macrophages and vessels in contact with human cancer cells and the fate of the latter.

**Figure 1:**
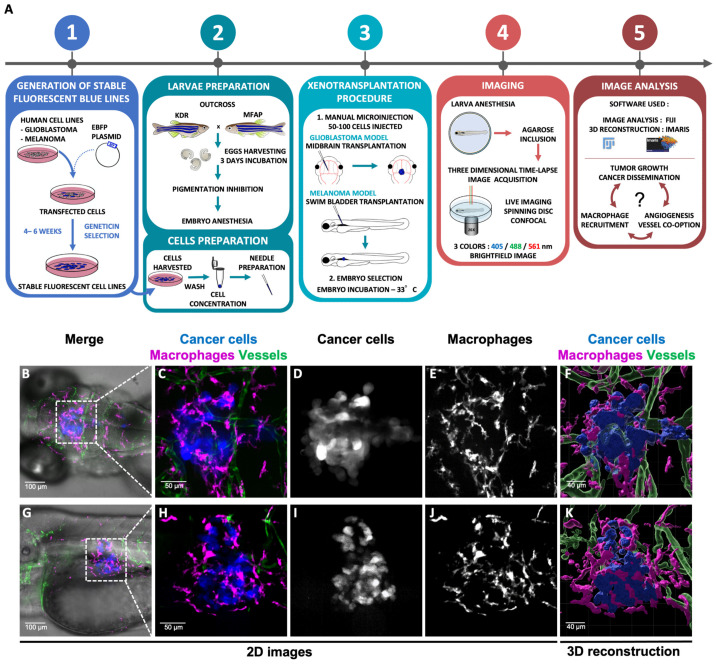
Experimental workflow and 3D glioblastoma and melanoma TME reconstruction. (**A**). The method is composed of five successive steps. Blue glioblastoma (GBM) and melanoma fluorescent human cancer cell lines (U87 and A375, respectively) were firstly generated (1). For this purpose, GBM cells and melanoma cells were transfected with a EBFP2 plasmid and selected over several weeks using Geneticin (1). Then, cells and embryos were prepared prior to transplantation (2). GBM cells were transplanted in the midbrain and melanoma cells in the swim-bladder of 3 dpf embryos (3). Imaging and time lapse were performed at 24 h or 48 h post transplantation (4). Image analysis was achieved using Fiji and Imaris software (5). (**B**–**K**) Each cell population is highlighted with a coded colour; cancer cells in blue or white, macrophages in magenta or white and vessels in green. (**B**–**E**) Visualization of GBM TME in the midbrain of zebrafish embryo at 4 dpf and 24 h post transplantation. Images represent a Z projection of 67 slices. (**G**–**J**) Visualization of Melanoma TME in the swim bladder of zebrafish embryo at 4 dpf and 24 h post transplantation. Images represent a Z projection of 74 slices. (**F**,**K**) A 3D reconstruction of tumours infiltrated by macrophages and vessels. (**E**,**J**) A high number of macrophages are recruited all around the tumour sites.

The general method consists of five steps synthesized in Figure 1A. The workflow includes the generation of the stable fluorescent blue lines, the embryo and cell preparation, the xenotransplantation procedure, and the image and video acquisition, followed by an analysis using Fiji and Imaris software. One day after transplantation, we visualized the recruitment of macrophages around the glioblastoma (Figure 1C) and melanoma tumour (Figure 1H).

### 3.2. Live Imaging of Interactions between Cancer Cells and Their Microenvironment

Using the Imaris Software, we reconstructed, in 3D, the tumour mass and the TME, including the macrophage population and vasculature (Figure 1F,K). From the 2D images and Z-stack, we observed that the recruited macrophages interact with cancer cells. However, only 3D visualisation allows us to characterize the physical orientation of macrophages around the tumour and their infiltration inside the tumour mass (Supplementary Videos S1 and S2). In addition, thanks to the time-lapse images, the dynamics of macrophages was monitored in 4D (Supplementary Videos S3 and S4). The long contact between the macrophages and glioblastoma cancer cells are visualized, suggesting an intense communication between the two types of cells (Supplementary Video S3). Recently, the pro-tumoral role of macrophages in zebrafish glioblastoma and melanoma models has been demonstrated [13,18]. Quantifying the number of macrophages and following their dynamics in time and space will unravel the steps leading to the evolution of the tumour and the appearance of cancerous metastases.

### 3.3. Advantage of 3D Reconstruction for Monitoring the Fate of Tumour Vasculature

**Figure 2:**
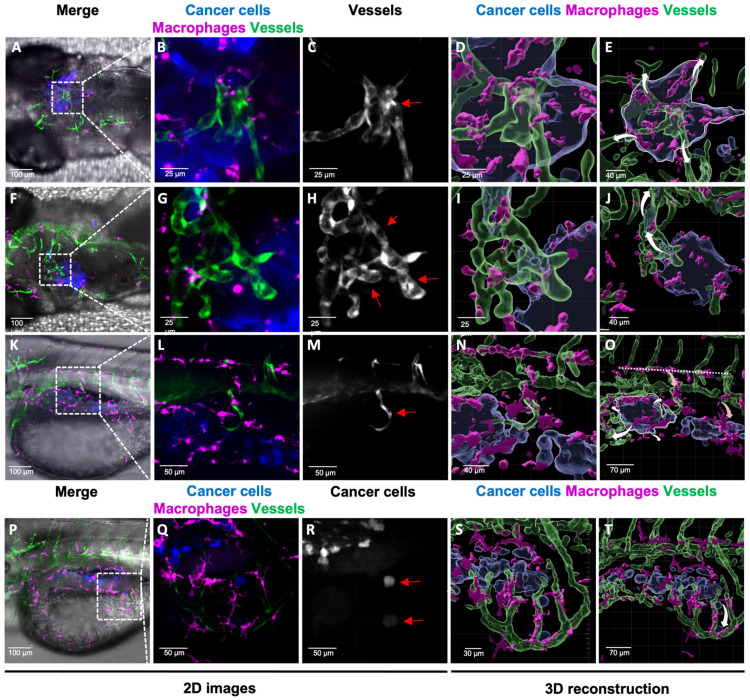
Analysis of 3D TME reconstruction and vessel dynamics: a predictive tool for tumour fate. (**A**–**T**) Each cell population is highlighted with a coded colour; cancer cells in blue or white, macrophages in magenta or white and vessels in green or white as shown in the images. The arrows in 3D reconstructions represent the prediction of tumour fate (white arrow) and macrophage fate (light-red arrow). (**A**–**E**) Z-stack projection (**A**–**C**) and reconstruction (**D**,**E**) of the glioblastoma TME live imaging 48 h after transplantation (**B**–**D**). The images show vessel dilatation in the centre of the tumour mass (**C**, red arrow). (**E**) The white arrow in the 3D reconstruction represents the prediction of tumour growth. (**F**–**J**) Z-stack projection (**F**–**H**) and reconstruction (**I**,**J**) of the glioblastoma TME live imaging 48 h after transplantation. Images represent a Z projection (Z = 30 slices) of the TME. (**H**) Vessel angiogenesis in contact with the tumour mass (red arrow). (**I**,**J**). A 3D reconstruction showing cancer cells migration through the vessels in the upper left corner. (**J**) The white arrow represents the predicted route of cancer dissemination regarding vessel localization. (**K**–**O**) Z-stack projection (**K**–**M**) and reconstruction (**N**,**O**) of the melanoma TME live imaging 24 h after transplantation. Images represent a Z projection (Z = 74 slices) of the TME. (**L**,**M**) Neo-angiogenesis is shown with a new vessel redirected inside the tumour mass (red arrow). (**N**) Macrophages are recruited at the tumour site following the new growing vessel. (**O**) The horizontal dashed line represents macrophages orientation outside the tumour site. The light-red arrows represent the orientation of the macrophage migration. The white arrows represent the predicted route of cancer cell dissemination regarding vessel localization. (**P**–**T**) Z-stack projection (**P**–**R**) and reconstruction (**S**,**T**) of the melanoma TME live imaging 24 h after transplantation. Images represent a Z projection (Z = 74 slices) of the TME. (**R**) In the images, two cancer cells detach from the primary mass following the vessels (red arrows). (**T**) The white arrow represents cancer cell migration and dissemination from primary mass.

We also investigated tumour vasculature evolution, a crucial player in the TME. One or two days after cancer cell transplantation, we observed angiogenesis and vessel co-option phenomena. We observed dramatic changes in vessel morphology upon contact with human glioblastoma and melanoma cells. The proximity to human glioblastoma cells directly influences the vessel dilation and shape (Figure 2B,C,G,H and Supplementary Video S4). The 3D reconstructions highlight the abnormal tortuosity of the vessels (Figure 2D) and their infiltration within (Figure 2I and Supplementary Video S4) the tumour. In the melanoma model, the cancer cells induce neo-angiogenesis (Figure 2L,M). The 3D reconstruction allowed visualization of the infiltrated new vessel inside the tumour mass (Figure 2N and Supplementary Video S5). Interestingly, in both cancer models, we observed vessel co-option (Figure 2J,T), with cancer cells beginning to migrate following the vessels as a dissemination route as described in the literature [2]. Thus, our method allows for a real-time visualization of tumour dissemination with the migration of cancer cells along the vessels. Simultaneously, the vessel morphology changes that promote tumour growth can be monitored. Real-time observation of vessel dynamics within the tumour is a valuable tool for the selection of new anti-tumour compounds such as anti-angiogenic treatments.

### 3.4. Importance of Macrophages in Tumour Angiogenesis

Figure 2N–O shows the evolution of macrophage shape and orientations in space, from horizontal to vertical orientation, which allows their migration following the vasculature inside the tumour niche. Thus, considering the importance of macrophages in tumour angiogenesis [19], the 3D reconstruction gives an insight of the crosstalk between these immune cells and vessels in the promotion of tumour progression.

## 4. Discussion

Here, we demonstrate the feasibility of modelling in 3D the dynamics of the TME at the cellular and tissular level. Thanks to our methods, we pave the way for a complex understanding of the TME architecture modification in vivo by visualizing the co-operation of specific cellular types such as macrophages and vessels, during tumour progression. This method can be implemented using other zebrafish transgenic lines to study other TME players such as neurons [20]. At the same time, this tool can be applied to study other cancer types, according to the different human cell lines used and the injection site. In this perspective, the 3D modelling of Patient-Derived Xenografts (PDXs) in zebrafish embryos would help clinicians with the treatment decision. Combining different colours in the same model allows us to study the involvement of different cellular types in one embryo, saving time by performing less experiments and thus decreasing experimental procedure costs. This is in line with the increased attention on the ethical guidelines. Finally, with the exponential interest in precision medicines, this time-efficient and reliable method, consisting in 3D modelling of the TME in zebrafish embryos, will represent a powerful tool to predict tumour fate in patients and to screen new efficient therapies.

## 5. Conclusions

Here, we report a cutting-edge methodology to visualize in 3D live imaging the interaction between the tumour microenvironment and cancer cells to study cancer fate. This methodology was validated in two different cancer models, the glioblastoma and the melanoma.

## Data Availability

The original version of the manuscript can be found at https://doi.org/10.1101/2021.09.23.461461.

## Supplementary Materials

The following supporting information can be downloaded at: https://www.mdpi.com/article/10.3390/cimb45110569/s1, Video S1: Navigation inside a 3D glioblastoma reconstruction to visualize macrophage recruitment and vessel organization. U87 human glioblastoma cells (blue) and its microenvironment such as macrophages (magenta) and vessels (green) are observed in the video. Human glioblastoma cells are transplanted in the midbrain region of zebrafish embryo at 3 dpf; the video represents the glioblastoma TME 24 h post transplantation. Macrophages are recruited all around the tumour mass and display close contact with glioblastoma cells, suggesting an intensive communication between both populations; Video S2: Navigation inside a 3D melanoma reconstruction to visualize macrophage recruitment. Human A375 melanoma cells (blue), macrophages (magenta) and vessels (green) are observed in the video. Human melanoma cells are transplanted in the swim bladder of a zebrafish embryo at 3 dpf. The video represents the melanoma TME at 24 h post transplantation. The navigation inside the TME 3D reconstruction allows us to visualize the massive presence of macrophages around and inside the tumour mass; Video S3: Macrophage dynamics in the glioblastoma tumour microenvironment. A 4D time-lapse reconstruction of U87 glioblastoma cells (blue) and macrophages (magenta). Human glioblastoma cells are transplanted in the midbrain region of zebrafish embryo at 3 dpf; the video represents the glioblastoma TME at 24 h post transplantation. The time interval between two images is 7 min. Two macrophages behavioural dynamics are highlighted. Macrophages in contact with glioblastoma cells (white arrowhead) tend to have a low motility profile, that allows for long lasting contact between the two populations. On the other hand, peripheral macrophages (yellow arrowhead), distant from the tumour, are more dynamic. Video S4: Glioblastoma TME dynamics. A 4D time-lapse reconstruction of U87 glioblastoma cells (blue) and their microenvironment, such as macrophages (magenta) and vessels (green). Human glioblastoma cells are transplanted in the midbrain region of a zebrafish embryo at 3 dpf; the video represents the glioblastoma TME 24 h post transplantation. The time interval between two images is 7 min. The reconstruction allows for the visualisation of vessels (white arrow) inside the core of tumour mass. Image acquisition allows us to visualize at the same time multiple events such as mitosis (yellow asterisk *) and macrophage migration along vessels (white arrowhead). The spatial position of the cancer mass in the TME is fundamental to understand the mechanistic role of vessels. Video S5: Navigation inside a 3D melanoma reconstruction to visualize vessel dynamics. Human A375 melanoma cells (blue), macrophages (magenta) and vessels (green) are observed in the video. Human melanoma cells are transplanted in the swim bladder of a zebrafish embryo at 3 dpf; the video represents the melanoma TME 24 h post transplantation. The navigation inside the 3D reconstruction allows us to visualize the dynamics of neo-angiogenic events. The spatial position of the cancer mass in the TME is fundamental to understand the mechanistic role of vessels. The video highlights the presence of a neo-growing-vessel inside the tumour mass located in the swim bladder where normally there is no vascular system. The specific localization of the vessel inside the tumour using the 3D reconstruction permits to predict the growth of the tumour itself.
